# Total venous nature of retinal deep capillary plexus inferred by continuity of prominent middle limiting membrane sign in optical coherence tomography

**DOI:** 10.1371/journal.pone.0257698

**Published:** 2021-09-21

**Authors:** Jialiang Duan, Minhao Li, Zhifeng Wu, Zhengwei Zhang

**Affiliations:** 1 Department of Ophthalmology, The Second Hospital of Hebei Medical University, Shijiazhuang, Hebei, People’s Republic of China; 2 Department of Ophthalmology, Affiliated Wuxi Clinical College of Nantong University, Wuxi, Jiangsu Province, People’s Republic of China; 3 Department of Ophthalmology, The Affiliated Wuxi No.2 People’s Hospital of Nanjing Medical University, Wuxi, Jiangsu Province, People’s Republic of China; University of California San Diego, UNITED STATES

## Abstract

This study aimed to theoretically identify the vascular nature of the deep capillary plexus (DCP) by examining patients presenting with both paracentral acute middle maculopathy (PAMM) and prominent middle limiting membrane (p-MLM) sign and p-MLM sign alone in spectral-domain optical coherence tomography (SD-OCT). A retrospective review of the medical records of patients with retinal vein or artery occlusion from two tertiary medical centers was performed. Consecutive patients with a clinical diagnosis of all categories of retinal artery occlusion (RAO) and retinal vein occlusion (RVO) (branch or central and ischemic or non-ischemic) who had undergone SD-OCT imaging from January 2015 to May 2020 were recruited and their p-MLM signs and PAMM lesions were assessed. We included 118 patients who presented with p-MLM sign with or without PAMM lesions. Amon them, 40 were female and 78 were male, with a mean age of 61.1 years. Of the 109 patients with both p-MLM sign and PAMM lesions, 23 had branch RAO, two had branch RVO, 67 had central RAO, 13 had central RVO, and four had a combination of central RAO and central RVO. All nine patients with the p-MLM sign alone had central RVO accompanied by cystoid macular edema. In all the enrolled patients, the hyperreflective lines of the p-MLM sign were continuous, regardless of the type of PAMM lesions. In conclusion, when PAMM and p-MLM sign are examined together, further proof regarding the possible complete venous nature of the vasculature of the retinal DCP might be speculated.

## Introduction

In 2013, the concept of “prominent middle limiting membrane sign” (p-MLM sign), which is a hyperreflective swelling line at the inner synaptic portion of the outer plexiform layer (OPL) on spectral-domain optical coherence tomography (SD-OCT), was introduced by Chu and associates [1]. Thus, we can infer that the plexiform layers are at increased risk of ischemia due to higher oxygen consumption rates and increased mitochondrial density [2]. However, because the p-MLM sign is considered synonymous with paracentral acute middle maculopathy (PAMM) and fails to account for the etiologic mechanism of deep capillary ischemia [3], references to it in published articles are gradually decreasing [4].

Conversely, multi-model imaging research of PAMM appears to be of great interest in recent years [5, 6], which helps clarify the blood flow and connection of different capillary plexuses in the retina. Due to the advent of optical coherence tomography angiography (OCTA), we can obtain depth-resolved imaging and detailed visualization of all three layers of the retinal capillary system in vivo compared with insufficient morphological information about the middle and deep capillary plexuses provided by conventional dye-based fluorescein angiography (FA) [7]. More importantly, OCTA can reliably identify communications between the three capillary networks via vertically oriented arterioles and venules [8, 9]. Despite this, the nature of blood flow through the retinal capillary system is greatly complex and debatable.

Nowadays, there are currently two major models for the organization of the retinal capillary plexuses: a hammock model (parallel organization) [10, 11] and serial organization [12, 13]. One of the main controversies between the two models lies in whether the deep capillary plexus (DCP) is entirely venous in nature [12] or consists of arterial and venous components [11]. Consequently, the present study primarily aimed to assess the vascular nature of the DCP by evaluating the characteristics of p-MLM sign and PAMM lesions in cases with retinal vascular occlusion diseases.

## Materials and methods

The Institutional Review Boards of Wuxi No.2 People’s Hospital, Affiliated Wuxi Clinical College of Nantong University and the Second Hospital of Hebei Medical University approved the protocol, and our study was conducted in accordance with the tenets of the Declaration of Helsinki.

This retrospective study recruited consecutive patients with a clinical diagnosis of all types of retinal artery occlusion (RAO) and retinal vein occlusion (RVO) (branch or central, ischemic or non-ischemic) who had undergone SD-OCT imaging (Optovue, Fremont, California, USA; Carl Zeiss Meditec, Dublin, CA; Heidelberg Engineering, Heidelberg, Germany) from January 2015 to May 2020 to assess PAMM and p-MLM sign from the Department of Ophthalmology, Wuxi No.2 People’s Hospital, Affiliated Wuxi Clinical College of Nantong University and the Second Hospital of Hebei Medical University. Moreover, patients who clearly presented p-MLM sign, with or without PAMM lesions, were included for further analyses. Written informed consent was obtained from each subject.

## Results

**Fig 1:**
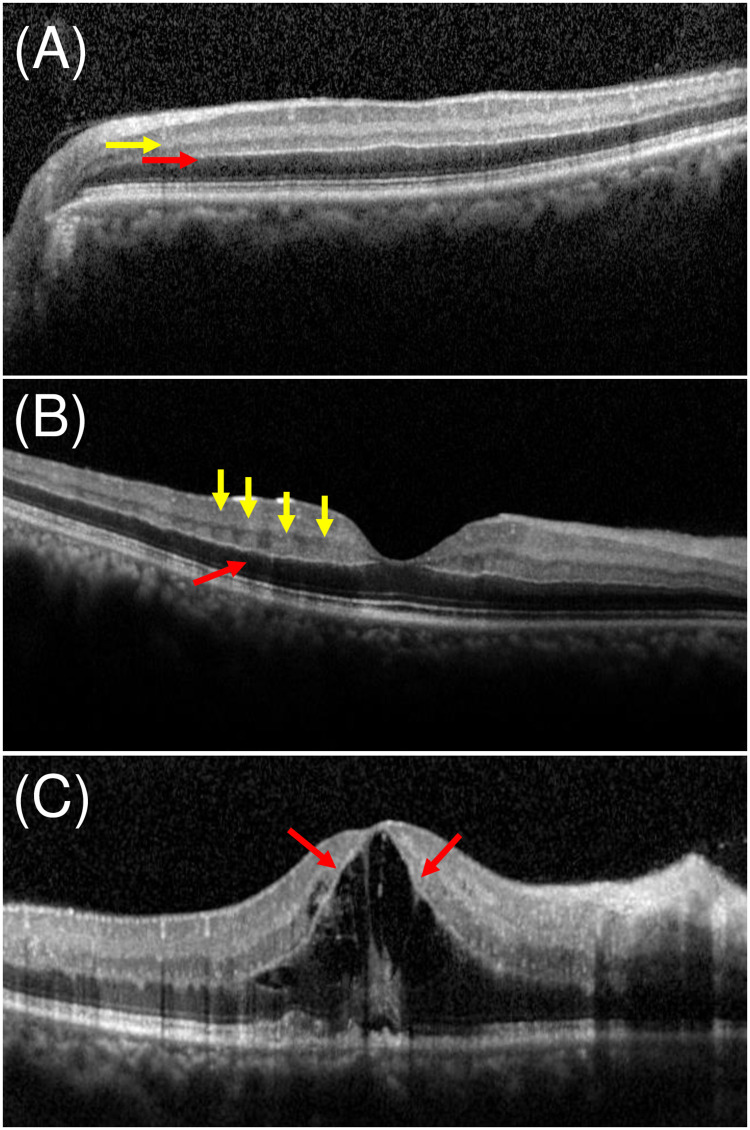
Three types of relationships between PAMM and p-MLM sign. Yellow arrows indicate PAMM lesions and red arrows indicate the hyperreflective line of p-MLM sign. A. Diffuse PAMM lesions and p-MLM sign. B. Skip PAMM lesions and p-MLM sign. C. p-MLM sign only. PAMM: paracentral acute middle maculopathy, p-MLM: prominent middle limiting membrane.

In total, 118 eyes of 118 patients (79 male and 39 female) with RVO and RAO who met the SD-OCT diagnostic criteria were included. The patients’ mean age was 61.1 (range, 9–90) years. Thirty-seven eyes had both diffuse PAMM and p-MLM sign (Fig 1A), 72 had both skip PAMM and p-MLM sign (Fig 1B), and nine had the p-MLM sign only (Fig 1C) (Table 1).

**Table 1:** Distribution of types of PAMM and p-MLM sign.

	Diffuse PAMM+p-MLM	Skip PAMM+p-MLM	p-MLM only
N (Female/Male)	37 (13/24)	72 (23/49)	9 (4/5)
Age (years)	66.19±10.65	60.01±14.66	49.11±10.22
Etiology (N)			
BRAO	8	15	0
CRAO	27	40	0
BRVO	1	1	0
CRVO	0	13	9
CRVO+CRAO	1	3	0

PAMM: paracentral acute middle maculopathy, p-MLM sign: prominent middle limiting membrane sign, BRAO: branch retinal artery occlusion, CRAO: central retinal artery occlusion, BRVO: branch retinal vein occlusion, CRVO: central retinal vein occlusion.

**Fig 2:**
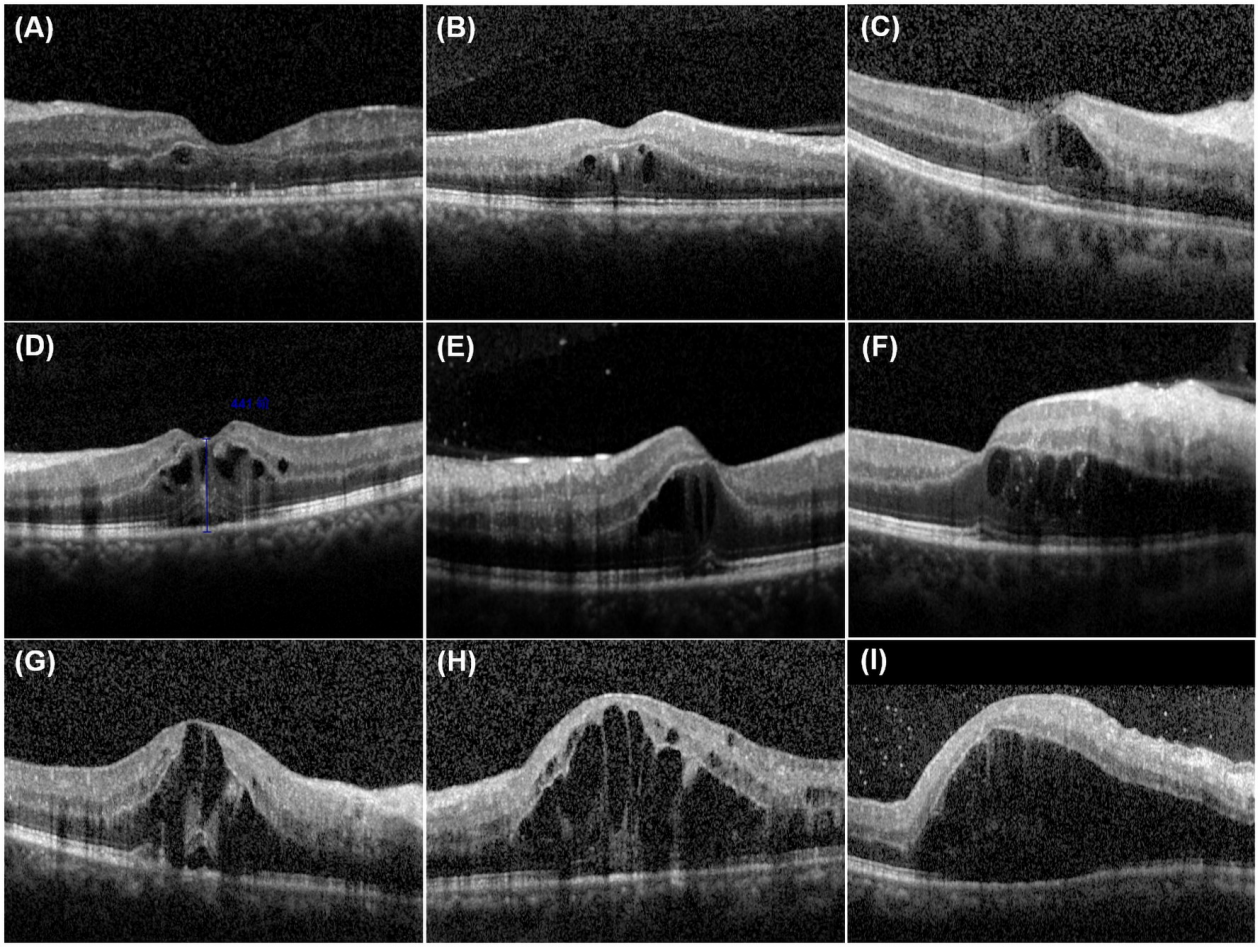
Nine cases with p-MLM sign alone. They all had central retinal vein occlusion accompanied by gradually aggravated cystoid macular edema (from A to I). p-MLM: prominent middle limiting membrane.

**Fig 3:**
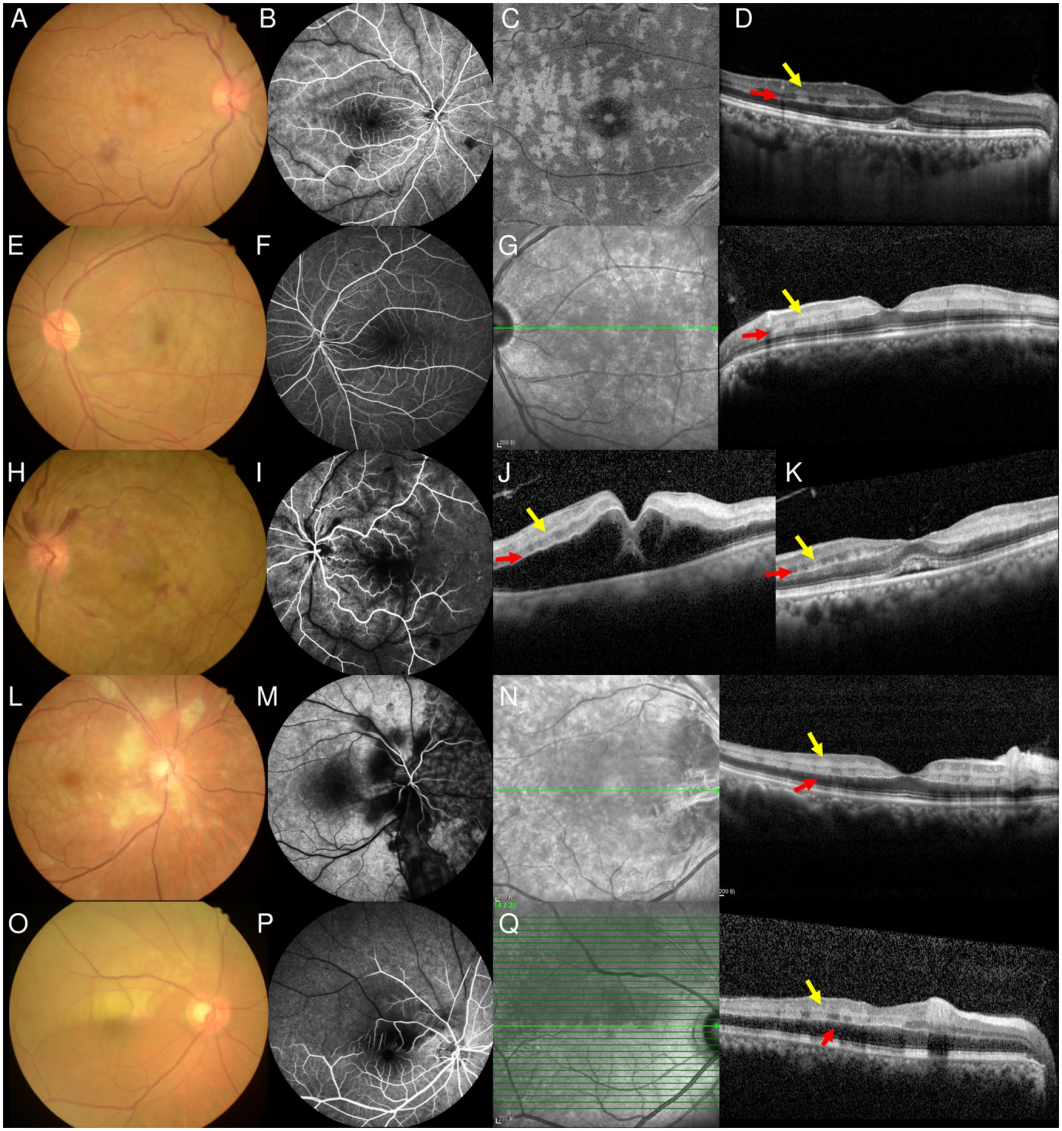
Multimodal imaging findings from the patient with retinal ischemic diseases. Line 1: A 47-year-old woman diagnosed with CRVO (A, B); en-face OCT reveals perivenular fern-like PAMM lesions (C); OCT reveals skip PAMM lesions (yellow arrow) and continuous hyperreflective line of the p-MLM sign (red arrow) (D). Line 2: A 55-year-old man diagnosed with CRAO (E, F); OCT reveals skip PAMM lesions (yellow arrow) and continuous hyperreflective line of the p-MLM sign (red arrow) (G). Line 3: A 69-year-old woman diagnosed with CRAO combined with CRVO (H, I); OCT before (J) and after (K) anti-vascular endothelial growth factor treatment all shows skip PAMM lesions (yellow arrow) and continuous hyperreflective line of the p-MLM sign (red arrow). Line 4: A 42-year-old man diagnosed with CRAO. Fundus photos reveal cotton wool spots around the optic disc (L); fluorescent angiography reveals delayed retinal artery perfusion and nasal choroidal perfusion (M). OCT reveals skip PAMM lesions (yellow arrow) and continuous hyperreflective line of the p-MLM sign (red arrow) (N). Line 5: A 63-year-old man diagnosed with BRAO (O, P). OCT reveals skip PAMM lesions (yellow arrow) and continuous hyperreflective line of the p-MLM sign (red arrow) (Q). OCT: optical coherence tomography, PAMM: paracentral acute middle maculopathy, p-MLM: prominent middle limiting membrane, CRVO: central retinal vein occlusion, CRAO: central retinal artery occlusion, BRAO: branch retinal artery occlusion.

In the 109 patients with both PAMM and p-MLM sign, 23 eyes with branch RAO (BRAO), two with branch RVO (BRVO), 67 with central RAO (CRAO), 13 with central RVO (CRVO), and four with CRAO+CRVO were observed. All nine patients with p-MLM sign alone had CRVO accompanied by cystoid macular edema (Fig 2). Interestingly, the hyperreflective lines of p-MLM sign were continuous in all patients (Fig 3).

## Discussion

Generally, large arterioles and venules are inherently connected via a capillary plexus; however, the accuracy of the conformance of the superficial, intermediate, and deep retinal capillary plexuses with the model of arterial inflow and venous outflow has not been completely clarified. In the beginning, two major models for retinal arteriolar supply and venular drainage have been proposed in recent years via novel noninvasive imaging technology in vivo: the hammock model [8] and the serial model [14, 15].

The hammock model states that all three retinal capillary plexuses have their own arteriolar supplies and venular drainage and form independent neurovascular units within the superficial, intermediate, and deep retinal layers [8]. Hence, this model suggests independent neurovascular control in different capillary layers according to the layers’ neuronal needs [10], especially in the inner plexiform and outer plexiform layers. Furthermore, the plexiform layers have mitochondria-rich synapses and high metabolic demand, increasing arteriolar blood supply. This model was supported by studies on the primate retinal vasculature development [16] and projection-resolved optical coherence tomography angiography study [8, 17]. For example, Nesper et al. [17] studied the hemodynamic response of retinal superficial capillary plexus (SCP), middle capillary plexus (MCP) and DCP in dark adaptation and flicker stimulation. In addition, the results provided evidence for neurovascular coupling in the retina under different light conditions, supporting independent differential regulation at the different plexuses.

Conversely, the serial model proposes the restrictions of arteriolar inflow to the superficial capillary plexus and the MCP and of venous drainage of the parafovea to the DCP. This model implies that each capillary plexus does not function as an independent neurovascular unit. Hence, this suggests that the DCP acts predominantly as a venular outflow tract for the entire retinal microvasculature and is filled with deoxygenated blood. This model was also supported by optical coherence tomography angiography and high-resolution confocal microscopy studies [12, 18, 19]. Besides, some authors found that, in 23 eyes with retinal vein occlusion, all 101 collateral vessels were found to course through the retinal middle and deep plexus [20]. More interestingly, Fragiotta et al. [21] presented the findings of an optical coherence tomography angiography case study in which they displayed remarkable vascular flow bridging the schitic cavities in the inner nuclear layer of a patient with X-linked juvenile retinoschisis. These pathological phenomena further support a serial arrangement of the retinal capillary plexuses.

Although these two models are simple and clear and indeed in accordance with certain clinical and experimental observations, they cannot explain all conditions. More recently, after continuous research and updates, evidence supports the improved the parallel model of retinal capillary organization as a composite network of complex horizontal and vertical interconnections. Consequently, some aspects of the two models were balanced and brought closer to a middle ground, known as the hybrid or mixed model [11, 13]. In the eyes of former supporters of the hammock model and serial model, they agree to contain arterioles and venules in the SCP and MCP; nevertheless, the vascular nature of the DCP, whether it is entirely venous [12], or both arterial and venous [11], remains the main controversy.

**Fig 4:**
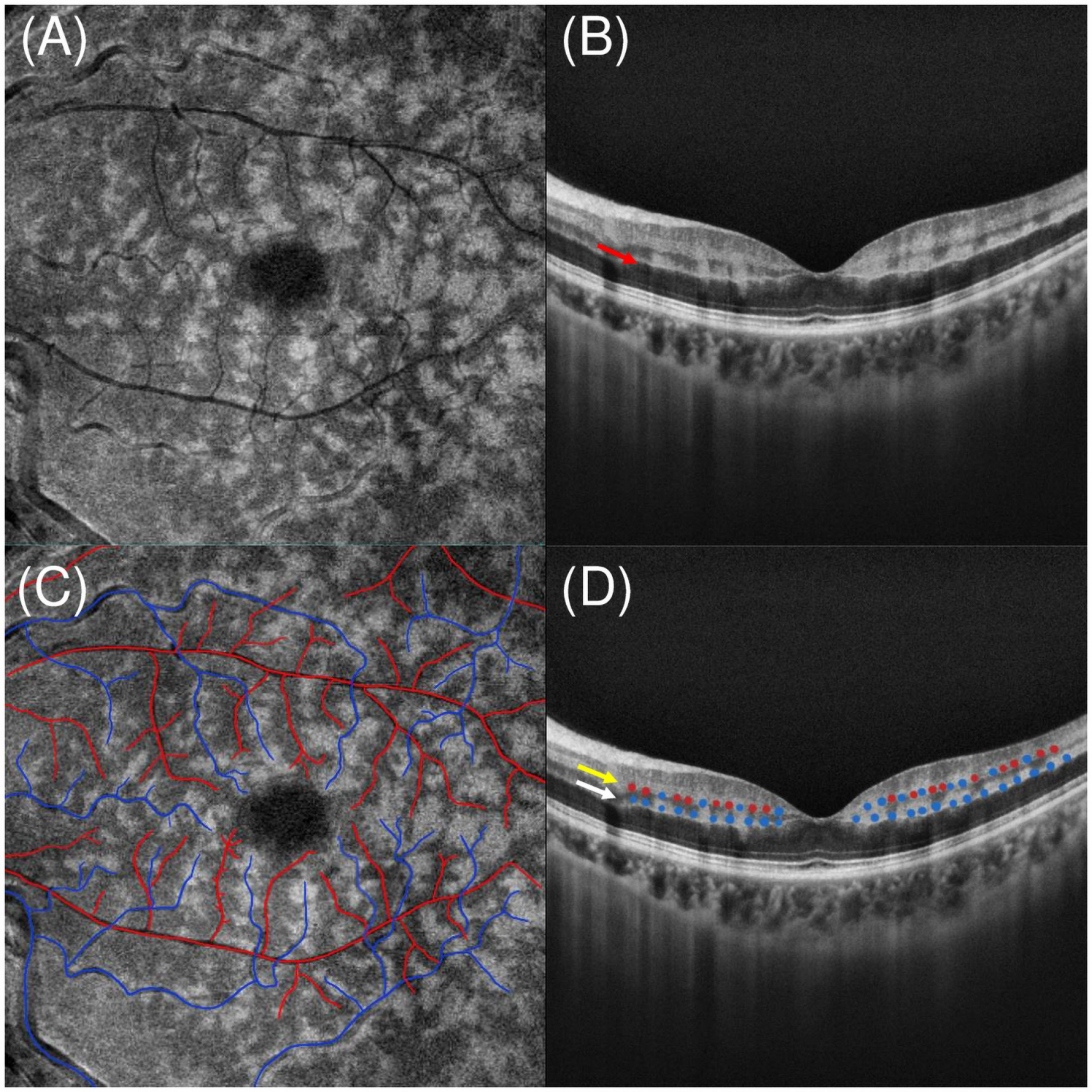
Speculated model of the vascular nature of MCP and DCP. OCT findings from a patient with central retinal vein occlusion combined with central retinal artery occlusion. En-face OCT segmented at the level of the inner nuclear layer and with vascular mapping illustrates a remarkable and precise perivenular distribution of fern-like PAMM with periarterial sparing (A). Cross-sectional OCT B-scan demonstrates skip PAMM lesions and continuous hyperreflective line of the p-MLM sign (red arrow) (B). In panel C (the same image as A), arterioles colored in red and venules colored in blue. Similar to en-face OCT, skip PAMM lesions from OCT may reveal that the nature of the MCP (yellow arrow) is both venous and arterial with alternate arrangement (D; arterial blood supply indicated in red dots and venous blood supply indicated in blue dots). However, the continuous hyperreflective line of the p-MLM sign might suggest that the nature of the DCP (white arrow) is purely venous according to our speculation (D; venous blood supply indicated in blue dots). OCT: optical coherence tomography, PAMM: paracentral acute middle maculopathy, p-MLM: prominent middle limiting membrane, MCP: middle capillary plexus, DCP: deep capillary plexus.

In our study, when the p-MLM sign and PAMM lesions were studied together, we discovered an interesting phenomenon that has not been previously evaluated. Specifically, we observed the continuity of the hyperreflective line of the p-MLM sign, regardless of the type of PAMM lesions, and this phenomenon was confirmed in all our patients (Fig 3). According to the concept of misery perfusion [22, 23], a decrease in oxygenation would preferentially affect the efferent component of the retinal circulation more than the afferent component. The pathophysiology of PAMM is considered to be deep vascular complex hypoperfusion or ischemia, considering that the oxygen supply of the inner nucleus layer was derived from both the MCP and DCP [24]. In both parallel and serial models (or in recent hybrid model), the MCP is considered to be interconnected by numerous vessels to both the arteriolar and venular aspects. Thus, the hypoperfusion of the deep vascular complex can cause multifocal skip lesions that co-localize with the perivenular pole, which manifests as skip PAMM lesions on SD-OCT B-scans or perivenular fern-like PAMM lesions on en-face OCT segmentation (Fig 4A and 4B) [25–27].

Due to the absence of direct blood supply from the retinal vessels, the oxygen supply of the inner synaptic portion of the OPL is mainly obtained from the oxygen diffusion from the DCP and choriocapillaris [2]. As the continuity of the p-MLM sign’s hyperreflective line shows inconsistency with that of the PAMM lesions (continuous or discontinuous), we infer that the oxygen saturation at the level of the inner synaptic portion of the OPL may be relatively uniform, which further implies that the oxygen saturation of the DCP should be uniform as well. Admittedly, the diffusion length of oxygen is often considered to be approximately 150 μm in the tissue under physiological conditions [28] and capillary spacing in the DCP is only about a third of this. However, when the retina is under hypoxic conditions, the diffusion distance for oxygen will likely diminish if the intracapillary pO2 decreases, and the inner synaptic portion of the outer plexiform layer is vulnerable to hypoperfusion due to its high oxygen demands [2]. In addition, if the blood supply of the line of p-MLM primarily comes from the dispersion of MCP, it would show an intermittent form as skip pattern PAMM. However, the lines of p-MLM of more than 100 cases in our study are all continuous. Therefore, this suggests that the nature of DCP could not be both venous and arterial (as illustrated by the alternate blue and red dots in Fig 4D), which have different oxygen saturation levels, but is entirely venous (as illustrated by completely blue dots in Fig 4D) [12]. Thus, when milder retinal hypoperfusion occurs, the whole level of the inner synaptic portion of the OPL (near the outer part of the DCP) and the inner nucleus layer tissue near the vein first experience ischemic edema, manifested as a continuous hyperreflective line, p-MLM sign only, or p-MLM sign with skip PAMM lesions. With the further aggravation of retinal ischemia, skip PAMM lesions can progress to a diffuse pattern.

However, the development of the p-MLM sign alone in cases with retinal vascular occlusion diseases is rare. In the present study, only nine patients exhibited the p-MLM sign alone. Interestingly, all of these patients had RVO with some degree of macular edema, and the p-MLM sign was mainly at the epicenter of the macular edema (Fig 2). In some patients, the p-MLM sign disappeared after macular edema was resolved (such as after anti-vascular endothelial growth factor administration). We hypothesize that when macular edema occurs in the outer retina, the uniform diffusion supply of oxygen from the choriocapillaris to the OPL may be disturbed by the distance created by the retina edema or cystoid changes. This was supported by the appearance of the p-MLM sign alone, which was always situated at the peak of the macular edema and gradually diminished with decreasing retinal thickness.

The inner synaptic portion of the OPL is thought to be rich in mitochondria [2] and has high oxygen demands. Thus, some researchers have speculated that DCP that supplies the OPL could not be composed of only venous blood, which carries lower oxygen [10]. However, the inner synaptic portion of the OPL is considered to be located at the functional anteroposterior watershed position between the DCP and choriocapillaris [2], which is not supplied by the DCP alone [29]. Therefore, the oxygen supply of the outer plexiform layer, located close to the DCP, may have developed a delicate balance between the retinal and choroidal circulation. Furthermore, no clear boundary demarcates which layer is supplied by retinal or choroidal circulation [29]. Based on our results, the p-MLM sign without PAMM lesions in RVO cases revealed that the oxygen supply of this part of the retina was fairly vulnerable to the choroidal oxygen supply, which was disturbed by the increased distance caused by macular edema and disappeared after macular edema was resolved. Further, if DCP had both artery and venous blood supply, it should have exhibited skip patterns in p-MLM sign similar to the skip PAMM lesions. However, none of our p-MLM cases exhibited skip patterns.

Our study has some limitations. First, one of the main limitations is that our conclusion is not based on the direct measurement of oxygenation of the DCP. Although Yu et al. [29] conducted substantial research on the oxygen partial pressure of the retina; unfortunately, most of their research focused on the “vertical” oxygen partial pressure between different retinal layers, rather than the “horizontal” oxygen partial pressure at different positions of the same retinal layer. Reports on the oxygen partial pressures at different positions on the same layer are limited, which may be because it is difficult and possibly inaccurate to directly measure oxygen partial pressures at different DCP positions. As observed in fern-like PAMM, the difference between different regions (hypoxia vs. no hypoxia) is only a few microns; however, the tip size of an oxygen-sensitive microelectrode is only approximately 1 μm [30]. In the future, it is necessary to directly measure the oxygen partial pressure of DCP with more advanced methods to determine its vascular nature. Most recently, a study indicated that the DCP of the retina is a purely venous plexus using high-resolution confocal microscopy in 16 normal human donor eyes [12]. Therefore, our study at least provides additional evidence using OCT scanned in vivo. Second, due to its retrospective design, the follow-up evaluation with ancillary imaging of most patients was not available. Third, not every subject had undergone a fluorescein fundus angiography examination; hence, we cannot accurately distinguish between ischemic and non-ischemic RVO cases.

## Conclusion

The present study examined over 100 patients with retinal vasculature occlusion by evaluating the presence and patterns of PAMM lesions and the continuity of the hyperreflective line of the p-MLM sign might reinforce the entirely venous nature of the DCP.
